# Surface Texture Analysis of Hardened Shafts after Ceramic Ball Burnishing

**DOI:** 10.3390/ma12020204

**Published:** 2019-01-09

**Authors:** Stefan Dzionk, Bogdan Scibiorski, Wlodzimierz Przybylski

**Affiliations:** Faculty of Mechanical Engineering, Gdańsk University of Technology, G. Narutowicza Street 11/12, 80-933 GDAŃSK, Poland; bogdan.scibiorski@pg.edu.pl (B.S.); wlodzimierz.przybylski@pg.edu.pl (W.P.)

**Keywords:** surface geometric structure, finishing by ball burnishing, hardened steel

## Abstract

This article represents the results of testing the surface condition of shafts manufactured by the burnishing process. The shafts with a hardness of approximately 62 HRC (Rockwell C). were burnished with a ceramic ball (Si_3_N_4_), where the force range was controlled by the means of a hydraulic system. The machining process consisted of hard turning shafts with cubic boron nitride (CBN) inserts, followed by burnishing with the use of various machining parameters, such as feed and force. The research focused on the examination of burnished surfaces, which was conducted for various structures after hard turning, and with variable burnishing parameters. The results obtained for the decrease of roughness are presented as the relation between the parameters Rat/Rab, which is approximately 2, while for Rpkt/Rpkb, it is around 3.7, respectively. Sat/Sab is around 2, while Spkt/Spkb is around 3.5 (where an index denotes the t-surface after turning, and the index b-surface after burnishing). The structure of the surface after burnishing and turning is described with roughness parameters, as well as with the photographs of the specimen surface, and the bearing area curve.

## 1. Introduction

**Figure 1:**
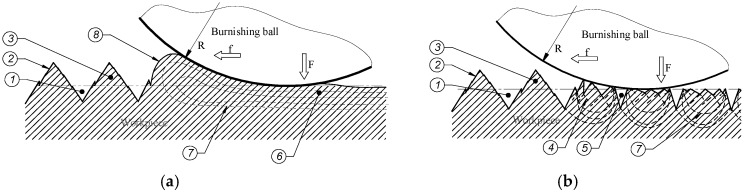
Schematic drawing of a burnishing process: (**a**) Soft materials; (**b**) hard materials, 1—valley of roughness, 2—surface profile, 3—peak of roughness, 4—slip plane, 5—deep valley after burnishing, 6—zone of material deformation, 7—zone of plastic and elastic deformation, 8—wave of material in front of the burnishing tool.

Durability and reliability are desirable features of today’s manufactured parts. These features depend, among others, on the properties and surface structures that are obtained as a result of the manufacturing process [1,2,3]. The surface structure also affects the useful properties such as wear and fatigue strength [4,5,6,7,8]. Obtaining an appropriate surface structure is usually associated with applying additional machining operations. An increase of quality and production capacity, and a reduction of costs necessitates the grouping of manufacturing operations. The components produced in 6–8 IT (International Tolerance—accuracy grade ISO 286) require an application of finishing operations. In this case, grinding is generally used, but this process requires an additional processing step. In order to reduce the number of manufacturing operations, burnishing is often used as a finishing process. This processes may be combined in one hybrid operation [9]. The burnishing process consists of plastic deformation of a surface by rolling or sliding burnishing tools. As a result of this process, the surface roughness decreases, and the surface layer strengthens [10,11,12,13]. This process can be performed with a lathe, but usually, it is combined with the shaping turning operation. Burnishing is most often used to processing soft materials whose hardness is below 40 HRC (Rockwell C). This process is well-known and described in the literature [14,15,16]. During burnishing, the soft material is deformed, not only on the surface, but also in the surface layer, at a significant depth. The literature [17,18] describes this process as being comprised of three stages:

In the first phase, (the initial stage), the burnishing tool exerts a considerable pressure on the peaks of the surface irregularities, creating plastic deformation by sliding occurring on the planes of the inner structure grains of a material. The increase of the pressure causes the deformation of the roughness peak surfaces, which cause a partial smoothing of the surface. In the second phase, a further increase of pressure of the burnishing tools against the surface causes the deformed material to move in the direction of the empty spaces near the tool contact point. The effect of this phenomenon consists of raising the valleys and decreasing the peaks of the burnished surface towards the surface of a tool. In the third (final phase), the burnished material fills the empty spaces on the burnished surface, and it moves along the contact area towards the front part of the tool. During the burnishing process, the tool moves forward along the surface of the worked material. In this case, the movement of the tool along the surface, and high pressures cause the outflowing of the material in front of the tool. The outflowing material creates an accumulation on the surface, which is displaced by front part of the moving tool. This formation is usually called a wave. This phenomenon is noteworthy, because during the process, the wave may be pulled under the burnishing tool, causing a defect on the surface of the workpiece. It can lead to the deterioration of the machined part’s suitability. The range of deformation of the material is quite high, and changes in the structure of the surface layer occur at a significant depth. A schematic drawing presenting the deformation during this type of machining is shown in Figure 1a.

Machining hard materials (hardened steel, cast iron alloys whose hardness exceeds 40 HRC) is a very efficient and cheap subtractive method. Tools for machining hard materials, for example, cutting inserts (CBN—cubic boron nitride, for machining hardened parts of up to 65 HRC) [19,20], are available in the market; however, obtaining the desired surface roughness parameters is not always possible. In this case, the surface can be improved by burnishing; however, this process has a different course than in case of burnishing soft materials [18,21,22,23,24]. The deformability of material is limited, and the smoothing of surface roughness occurs through material sliding, only within the roughness zone (there are generally no changes in the subsurface layer). In the view of the considerable resistance of hard materials for plastic deformation, only the first-stage phenomena of the above-mentioned deformation process occur in the deformation process [25,26]. During burnishing, hard materials do not allow for the formation of a material wave on the surface in front of the processing tool. In such a case, the sliding of material in the roughness zone during the burnishing process causes only a decrease of the range of the roughness zone. During machining of very hard steel (62–65 HRC), the deformation range is unable to complete the filling up of the valleys on the surface that are created from the preceding process, e.g., turning, which is opposite to the full deformation in the roughness zone that occurs during the burnishing of soft materials. As a result of this phenomenon, traces from the previous process that are not removed by burnishing are left on the surface of the processing parts. A schematic drawing of the process can be seen in Figure 1b.

Burnishing of hard materials is difficult, and it is believed that this is an ineffective process. The advantages of this process is that it can be carried out, together with turning, using the same workplace. This will shorten the machining time, because the workpiece does not have to be moved for setting and clamping. Thus, the quality of the surface obtained in this process is sufficient for usage under rolling bearings, the joint of the hub shaft, etc.

The paper discusses the surface structure results of burnishing hard shafts by the means of ceramic balls which are pressed by hydraulic system and lubricated with processing fluid.

## 2. Experimental Details

**Figure 2:**
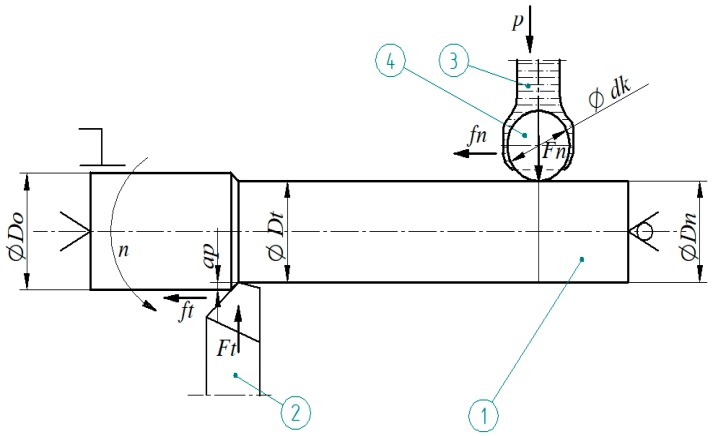
Schematic drawing of a machining process: 1—machined shaft, 2—tool for hard turning, 3—processing liquid, 4—burnishing ball, *ft*—turning feed, *ap*—depth of turning, *Do*—diameter of the processed shaft, *Dt*—shaft diameter after turning, *Dn*—shaft diameter after burnishing, *Fn*—burnishing force, *fn*—burnishing feed, *p*—pressure of the processing liquid, *dk*—diameter of the burnishing ball.

**Figure 3:**
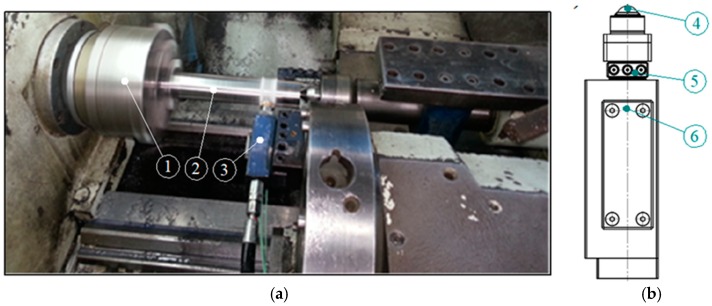
Burnishing a shaft on a computerized numerical control (CNC) lathe: (**a**) View of the burnishing process; (**b**) schematic drawing of a hydrostatic burnishing tool with a “Kisttler” sensor, 1—a spindle with a chuck, 2—the workpiece, 3—a burnishing tool, 4—the burnishing ball, 5—a force sensor, 6—a shank with hydraulic pressure.

**Figure 4:**
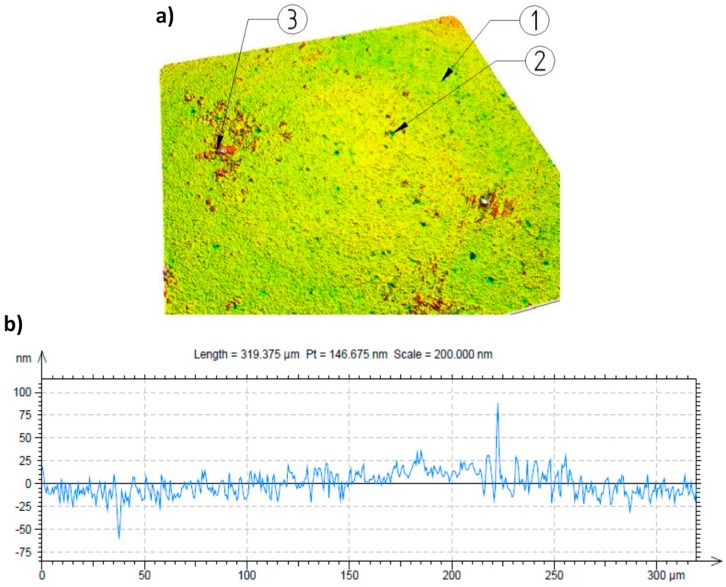
Surface structure of the burnishing ball made from Si_3_N_4_: (**a**) Surface photograph; (**b**) surface profile, 1—surface structure, 2—small pores occurring on the surface, 3—additional material on the ball surface.

The research was carried out using the computerized numerical control (CNC) lathe type Razmer 2M-45-21/11. The tests were performed on hardened shafts, which were processed by turning, followed by burnishing. The schematic drawing of the process is presented in Figure 2, and a photograph of the process is shown in Figure 3. A special tool for burnishing was prepared, in order to implement the processing, where the burnishing force was being set up by the hydraulic system, and balls were placed in hydrostatic bearings (Figure 3b). Ceramic bearing balls of a diameter of 0.5” made of silicon nitride (Si_3_N_4_) were used as a burnishing element of the tool. The structure of the burnishing ball surface is shown in Figure 4, and its surface structure parameters are presented in Table 1. The hardness of these balls ranged from 1500–1800 HV compressive strength (800 °C) in the range of 1.2–5.2 GPa, and a fracture toughness of 1.8–6.5 MPa m^1/2^. The burnishing tool was steered and supplied by the hydraulic unit, generating pressure to 40 MPa. This pressure allows for the realization of an equivalent ball downforce of about 1.6 kN. Processing fluid was applied as an 8% emulsion of oil and water (commercial name: Hydrol R). The hard turning of the shafts was performed by insert CBN (WNGA 080408 WZ-LS3 TB 650, TaeguTec, Daegu, South Korea). The shafts were made from steel 1.1213 (C53). The properties of this material in its soft state are in Table 2. The shafts were treated by induction hardening at a depth of approximately 2 mm. The surface hardness after this heat treatment was 62 ± 2 HRC. The machining process parameters are presented in Table 3. Measurements of the surface structure of the tested shafts were made by using a confocal microscope “µsurf Exploler” by Nanofocus AG (Oberhausen Germany).

The machining conditions for turning and burnishing are presented in Table 3. The turning feed rates were selected in such a way as to differ from the value of the feed rates adopted for burnishing process. In this way, it would avoid the situation in which the pressing ball is moved on the trace of the turning tool. This is important for hard materials machining, because the deformation on the surface is small, and it is possible that the flattening of peaks would not occur sufficiently. Also, the same value of feed for turning and burnishing is not recommended in the literature [23].

During the burnishing of hard materials, the value of the feed rates is significantly smaller in relation to the values that are applied during turning, which generally enables a greater reduction of the surface roughness parameter values, e.g., Ra [17,19]. It should be noted that regardless of the above statements, the roughness reduction may significantly influence the features of the element of a burnishing tool that is applied, e.g., the profile radius, the state of the surface. In the experiment, a ball whose diameter was ½ inch was applied. The surfaces of the balls were examined with a confocal microscope. The results of these examinations are presented in Figure 4.

**Figure 5:**
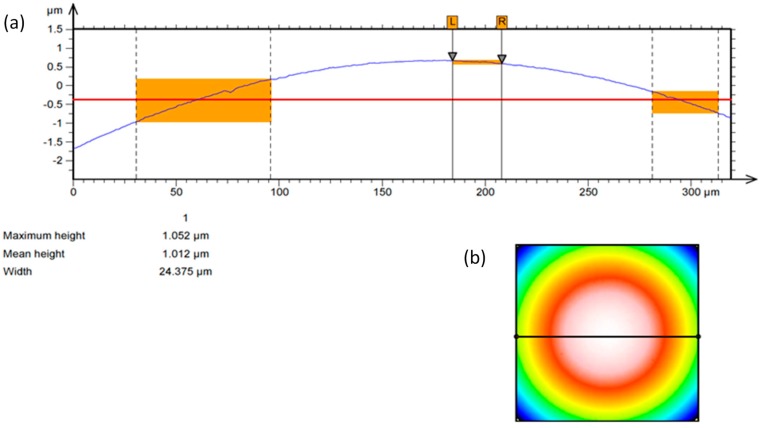
Contour of the Si_3_N_4_ burnishing ball: (**a**) Profile of the ball surface; (**b**) view of the surface of a sphere.

The characteristics of the ball contour are presented in Figure 5. The results of the examination of the roughness parameters of burnishing balls are presented in Table 1. For the ball diameter specified above, two values of the burnishing feed rate were defined in the research plan.

The adopted values of the burnishing feed rate were 0.06 mm/rev and 0.2 mm/rev. These values correspond to the above-mentioned assumptions for the selection of the burnishing feed value coefficient, i.e., they differ from the turning feed coefficient values *ft* (see Table 3). In the research, a feed value *ft* = 0.18 mm/rev was adopted, which was slightly smaller than the burnishing feed value *fb* = 0.2 mm/rev. The following combinations of operations: HT1+B1, HT1+B2, HT2+B1, and HT2+B2 were admitted for analyses. The operations following turning consisted of one-off burnishing operations (one pass of the burnishing operation following the turning operations). (Both hard turning and burnishing operations were performed on a CNC lathe, type Razmer 2M-5-21/11.)

## 3. Experimental Results and Discussion

The results of the research carried out according to the plan specified in Table 3 are presented in Table 4 and Table 5. The measurements were performed for the 2D (ISO 4287) and 3D (ISO 25178) structures. Comparing these results, significant differences in the measurement results between the 2D parameters Rz, Rv, and Rp, and the 3D parameters Sz, Sv, and Sp can be observed. The differences may be due to the removal of individual grains of workpiece material from the surface during hard turning. This phenomenon may confirm the occurrence of the individual valleys of considerable depth. The values of the valleys are larger than the highest peak within the measured area. The main objective of burnishing machining is the reduction of the heights of surface irregularities. This objective was reached because the burnishing results showed that the height parameters of the surface structure were reduced by approximately two-fold, which is shown in the results listed in Table 4 and Table 5.

**Figure 6:**
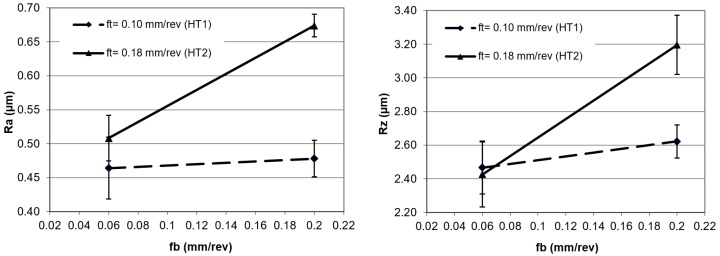
Surface roughness after burnishing; Ra and Rz are parameters as a function of feed rate.

**Figure 7:**
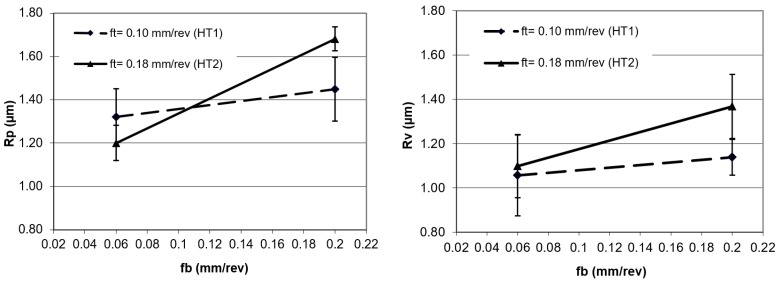
Surface roughness after burnishing; Rp and Rv are parameters as a function of feed rate.

Figure 6 and Figure 7 show the results of the surface structure changes after applying various burnishing feed rates. It can be noted that for the burnishing feed rate of 0.06 mm/rev, the effect of the process on the parameters obtained after turning was small for the Ra parameter, while this did not occur for the Rz parameter.

On the basis of the results, and from the analysis of the deformations of the peaks and valleys depicted in Figure 7, it can be observed that the deformations of the peaks were bigger when compared to the deformations of valleys. A low value of burnishing feed made that process stable. The scatter of the measurement results in Figure 6 and Figure 7 are presented in the standard deviation, shown as a range around the points of the graph.

**Figure 8:**
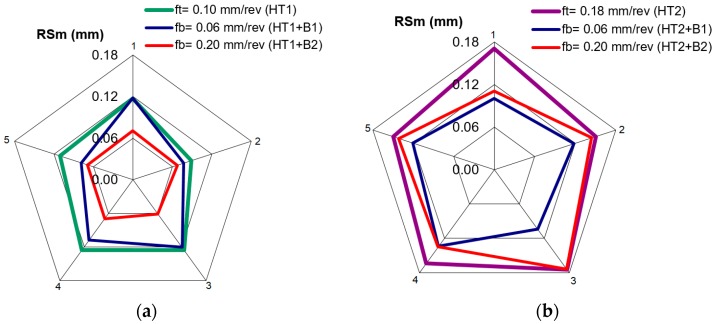
Value of the RSm parameter after turning and burnishing for five measurements: (**a**) for turning HT1; (**b**) for turning HT2.

The average width of the profile elements grooves RSm corresponds the approximate feed rate values that are used in the turning. Figure 8 shows the changes of the RSm parameter as a result of burnishing. A stable roughness determined by the RSm = 0.06 mm parameter was obtained on the surface of a turned sample with a feed of 0.10 mm/rev, after burnishing with a feed rate of 0.20 mm/rev. Such stability of the RSm parameter was not obtained for other turning and burnishing parameters. This may be due to the surface deformation of the hard materials, and traces remaining after the previous machining process. When the turning feed rate was 0.18 mm/rev and the burnishing feed rate was 0.06 mm/rev, the process was stabilized. In this case, the RSm parameter was not being reduced much, despite the fact that the burnishing feed was much smaller than in the above-mentioned case. It should be noted that in the above cases, the value of the turning feed was a multiplication of the burnishing feed rate. This relation between the machining parameters allows each groove to be burnished several times after turning, resulting in a process that is constant for the entire surface.

However, when the feed rate is not a multiplication of the burnishing feed rate, some grooves are burnished more times than the others, which causes variability in the surface parameters.

**Figure 9:**
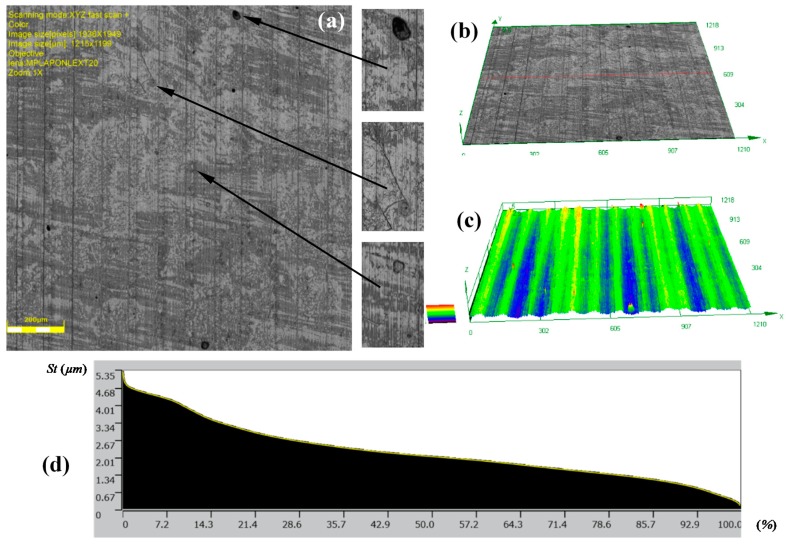
Topography of the surfaces produced in hard turning HT1: (**a**) photograph of a surface; (**b**) 3D view of a surface; (**c**) 3D surface profilograph; (**d**) bearing ratio curve.

**Figure 10:**
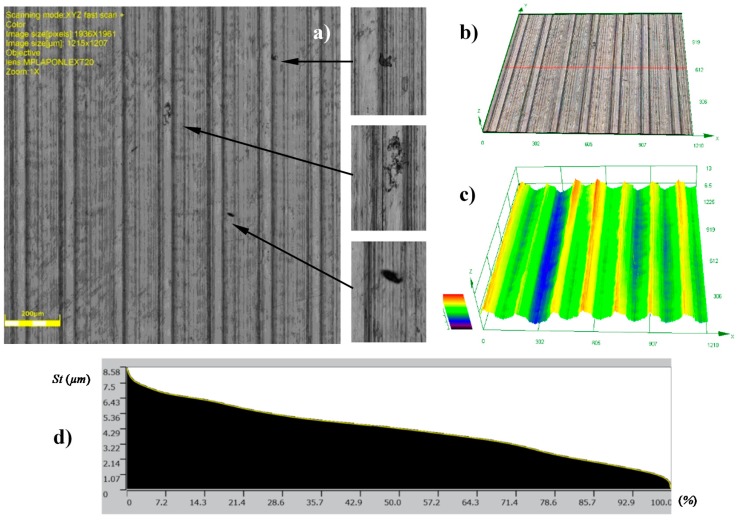
Topographies of surfaces produced in hard turning HT2: (**a**) Photograph of a surface; (**b**) 3D view of a surface; (**c**) 3D profilograph of the surface; (**d**) bearing ratio curve.

The topographies of the parts surfaces obtained after hard turning is presented in Figure 9 and Figure 10. A machining process with a feed rate of 0.1 mm/rev (code HT1) and 0.18 mm/rev (code HT2) yielded surfaces that were differentiated in terms of their structural parameters. The values of the 2D and 3D surface roughness parameters are presented in Table 4 and Table 5.

The surface structure features certain textures that are characteristic for surfaces after the turning process. The depths of the grooves in the structure are variable. The bearing ratio curves are very similar; hence, the structure of the irregularities is the same, although they have different roughness parameters. This is also confirmed by similar structures of the material peaks Mr1, and valleys Mr2. They are 12.84%, 91.30%, 14.81%, and 88.32%, respectively, for the core roughness Rk = 2.563 μm (code HT1) and Rk = 4.494 μm (HT2). The increase of the value of the turning feed preceding burnishing caused an increase in the values of the following roughness parameters: Ra, Rz, Rp, and Rv. Detailed results are presented in Table 4.

The analysis of 3D roughness parameters indicate a similar correlation to the previously presented data. Only the Sz, Sp, and Sv parameters had higher values after HT1 processing. If there are single peaks on the surface, the 3D surface measurements are more precise than the 2D measurements. In the analyzed case, isolated peaks and valleys expressed in the surface parameters are caused by surface flaws or inclusions (Figure 9a). A comparison of the Sp = 13.084 μm and Sv = 17.63 μm parameter values indicated that there were dents and chippings on the surface of the workpiece material. The observation of the 3D profile (Figure 9c) indicated the possibility of inclusions. Similar flaws have been observed after turning the HT2 (Figure 10a).

**Figure 11:**
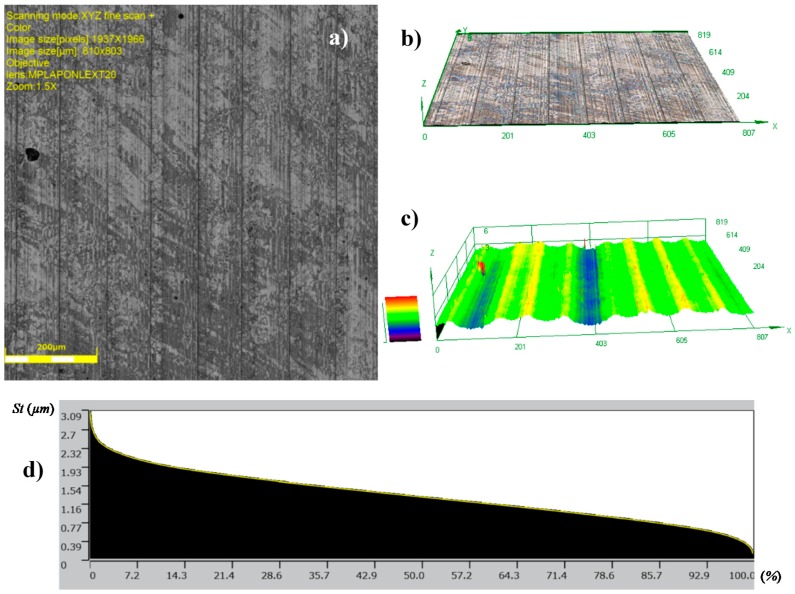
Topographies of surfaces after: HT1+B1: (**a**) Photograph of a surface; (**b**) 3D view of a surface; (**c**) 3D profilograph of the surface; (**d**) bearing ratio curve.

**Figure 12:**
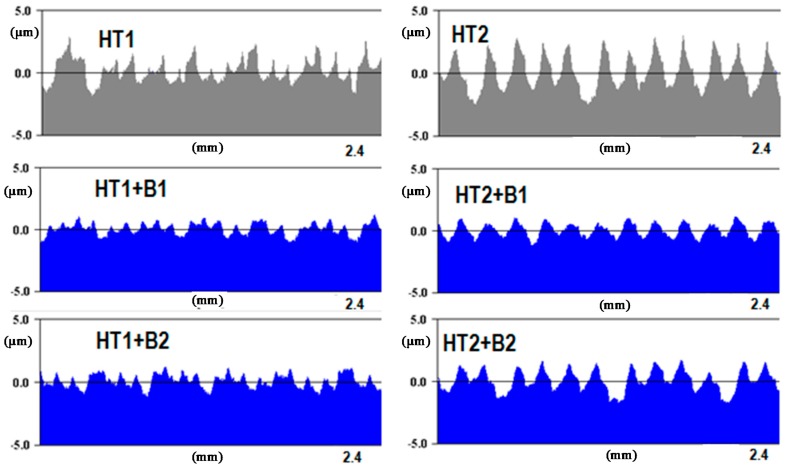
2D surface profiles obtained for different processing parameters determined by the code of the process.

Figure 11 shows the surface after burnishing with *fb* = 0.06 mm/rev. (code HT1+B1). In the result of machining, a reduction in the values of the surface roughness parameters (Table 4 and Table 5) was noted. In this case, the value of the Ra = 0.859 μm parameter obtained after turning (HT1) decreased to Ra = 0.416 μm after burnishing (HT1+B1), for 2D and 3D profile measurements, while the value of the Sa = 0.849 μm parameter obtained after turning (HT1) decreased to Sa = 0.416 μm after burnishing (HT1+B1). The surface featured clear traces of the preceding process that were associated with its parameters, especially the feed. It can be assumed that changes in the structure of the surface roughness occurred directly on the surface, causing the smoothing of surface texture peaks. This phenomenon has been explained in Section 1, when discussing the mechanism of burnishing hard materials (Figure 1b), where the movement of the burnished material is limited, and the burnishing loads are transferred by surface peaks. This process causes sliding, which deforms the peaks, decreasing surface irregularities at the same time. This deformation course is confirmed by 2D profiles (Figure 12) made by the contact profilograph method. A reduction of the aforementioned surface profile peaks is visible in this figure.

This reduction can also be seen in the changes of the values of the parameters that are associated with the bearing ratio material Rpk parameter. In comparison with the values of the average parameters of surface roughness, and subsequently, Ra and Sa, the Rpk and Spk values were reduced, similarly to other parameters of the geometrical structure. For example, the relation between Rat/Rab for variant HT1+B1, was approximately 2, while for Rpkt/Rpkb, it amounted to about 3.7. Sat/Sab was around 2, while for Spkt/Spkb, it amounted to about 3.5 (where index t denotes turning, and index b denotes burnishing). The value of the reduction of surface roughness parameter *Ra* for hard materials reached up to 2.5, and therefore, it is necessary to obtain low roughness parameter values after turning (in this case, for HT1 turning, the relation between the Rat/Rab parameters was approximately 2 for variant HT1+B1).

**Figure 13:**
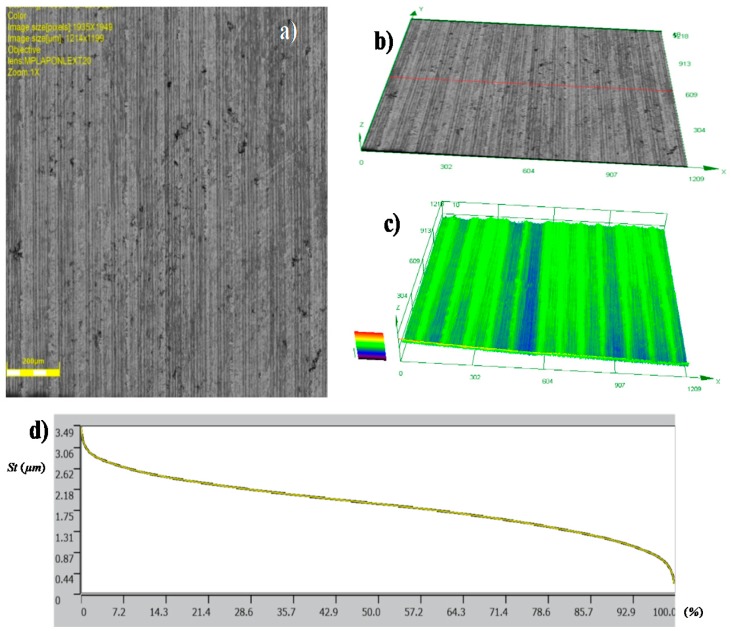
Topographies of surfaces after: HT1+B2: (**a**) photograph of a surface; (**b**) 3D view of a surface; (**c**) 3D profilograph of the surface; (**d**) bearing ratio curve.

The results of the surface roughness examinations after burnishing with the feed parameter 0.2 mm/rev (HT1+B2) are similar. Also, in this case, only the heights of the peaks decreased in the surface profile (Figure 13) and the surface featured visible traces of previous machining. The surface roughness parameter values did not significantly differ from the results obtained after burnishing the HT1 variant B1, e.g., Ra = 0.451 μm, and Sa = 0.446 μm (Table 4 and Table 5), and the relation between the values of the parameters of surface roughness after turning and the values after burnishing was also similar. This indicates that trebling the value of the burnishing feed did not result in a significant deterioration of surface roughness in relation to the surface obtained after burnishing in variant HT1+B1. In both variants, burnishing decreased the roughness parameters, and yielded a more favorable surface structure, as far as the functionality was concerned. This is confirmed by a negative value of the skewness profile parameters Rsk = −0.279 and Ssk = −0.171 (skewness, surface skewness, respectively) obtained for HT1+B2.

**Figure 14:**
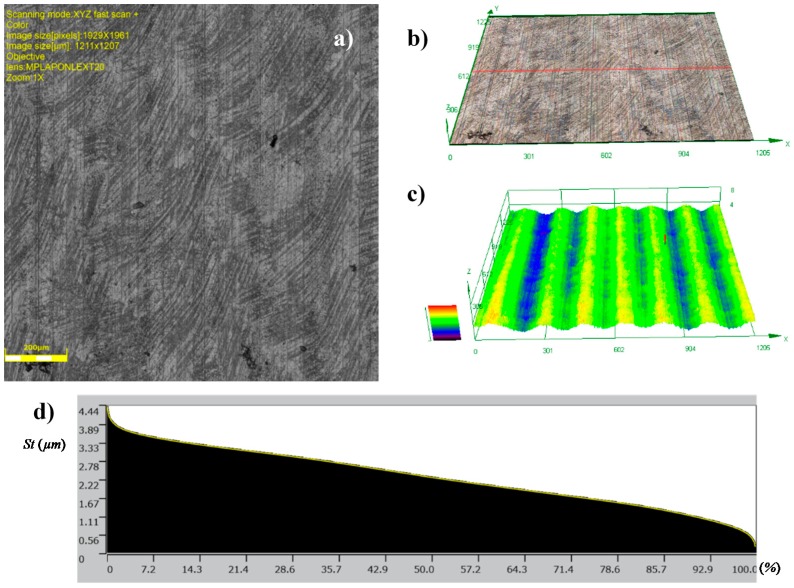
Topographies of the surfaces after: HT2+B1: (**a**) photograph of a surface; (**b**) 3D view of a surface; (**c**) 3D profilograph of the surface; (**d**) bearing ratio curve.

**Figure 15:**
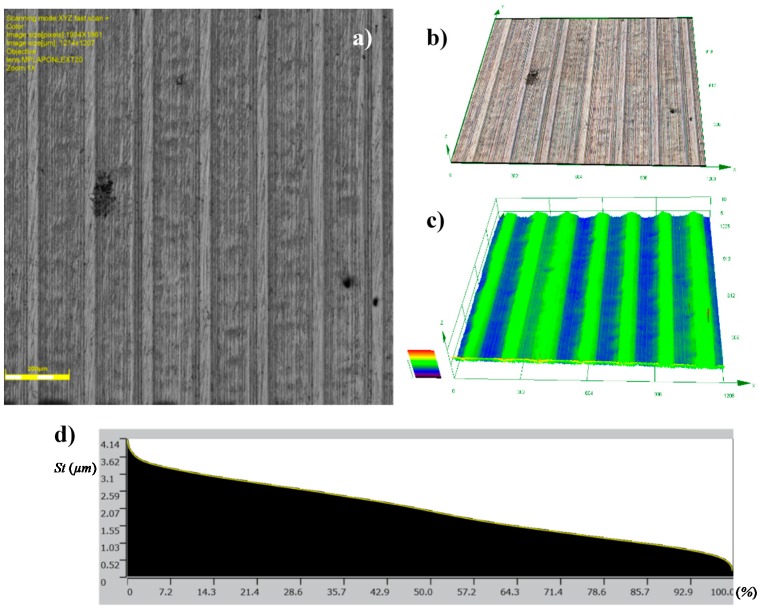
Topographies of surfaces after: HT2+B2: (**a**) photograph of a surface; (**b**) 3D view of a surface; (**c**) 3D profilograph of the surface; (**d**) bearing ratio curve.

Figure 14 and Figure 15 present the views of the surface after treatment with HT2+B1 and HT2+B2 parameters. Clearly, visible traces remaining on the surface after turning (feed *ft* = 0.18 mm/rev) can be seen in Figure 10, while the structure obtained after machining with lower burnishing feed value applied (*fb* = 0.06 mm/rev) also features traces left by the ball (burnishing element). This value of the feed parameter has a greater reduction of height surface peaks, as seen in Figure 9. Additionally, initial values of higher surface roughness parameters obtained after turning with feed *ft =* 0.18 mm/rev affected the final results of the surface parameters after burnishing (Table 4 and Table 5). It is possible to conclude that surface roughness parameter after burnishing depends more on the surface roughness obtained in the preceding process, and not the roughness that can be obtained by changing the burnishing parameters, especially feed *fb*. For example, the values of the parameters Ra = 1.457 μm and Sa = 1.522 μm, obtained after turning (HT2), changed to Ra = 0.716 μm and Sa = 0.728 μm after burnishing (HT2+B1), and Ra = 0.746 μm and Sa = 0.755 (HT2+B2).

A Comparison of the results for the HT1+B1 and HT1+B2 machining variants yielded similar values of the coefficients (the values surface roughness parameters after turning Rt related to the parameters obtained after burnishing Rb). The calculated values of Rat/Rab and Sat/Sab ratio amounted to approximately 2, whereas for variants of (case) HT2+B1 and HT2+B2, Rpkt/Rpkb and Spkt/Spkb they were 2.9 and 2.4, respectively. In this case, the higher value of the surface texture parameter changes associated with the bearing ratio, and the negative and zero values of the skewness profiles prove that an improvement of the functional quality of the surfaces has been achieved.

The obtained surface structures, and in particular, the material bearings ratio curve, give the possibility of using this burnishing method for surfaces used in hub joints, a tapper joint, a surface for rolling bearings, etc. Such a process should be used when dented abrasive grains cannot remain on the surface after the grinding process. This applies to cases where hard surface mating occurs in very extreme environments, i.e., high pressure, high temperature, difficult lubrication, etc., e.g., valves in the engine.

The surface characteristics presented in this article can be supplemented by information about the structural characteristics of the surface layer that is formed from the burnishing of hard materials. An article presenting these issues is under preparation.

## 4. Concluding Remarks

As a result of the surface burnishing after turning the hardened shafts to the value of 62 ± 2 HRC, a significant reduction in the surface roughness parameters was obtained. For example, the relation between Rat/Rab for feed rate turning at 0.10 mm/rev and feed rate burnishing at 0.06 mm/rev, was approximately 2, while for Rpkt/Rpkb, it was about 3.7, respectively, and Sat/Sab was around 2, while for Spkt/Spkb, it amounted to about 3.5 (where index t denotes turning, and index b denotes burnishing). In addition, it was observed that the deformation process of surface irregularities mainly takes place in the zone of the peaks. Deformations through slides in the peaks zone cause the valley to remain unfilled, and for traces to remain on the surface in the form of recesses, after the previous turning operation. The size of these traces of pre-processing can be limited by using small rates of turning feed, or small feed rates of burnishing. If the burnishing feed rate is much smaller than the rolling feed rate, then every trace after turning is deformed by repeated passing of the burnishing tool. The rolling feed rate has a greater influence on reducing the size of the above traces.

The curves of the bearing material ratios of the surfaces obtained by burnishing are similar to the curves obtained by other finishing treatments, e.g., grinding. These curves are characterized by a stable *Rk* parameter, which reduces their wear in frictional mating, and has cavities that can be used to store the lubricant. The negative values of the profiles parameters’ asymmetry provide good bearing ratios for the surfaces of these parts (Rsk, Ssk), which justifies the application of the burnishing process to manufacturing mating sliding parts.

## Figures and Tables

**Table 1:** Selected parameters of 2D and 3D surface roughness for the burnishing balls made from the Si_3_N_4_ ceramic.

Parameters According to ISO 4287						Parameters According to ISO 25178					
2D Parameters	Sample			Medium Value	Standard Deviation	3D Parameters	Sample			Medium Value	Standard Deviation
	1	2	3				1	2	3		
*Ra* (nm)	8.04	6.27	5.73	6.68	1.21	*Sa* (µm)	0.009	0.009	0.011	0.010	0.001
*Rz* (nm)	70.65	45.71	51.7	56.02	13.02	*Sp* (µm)	0.312	0.187	0.293	0.264	0.067
*Rt* (nm)	130.25	67.86	68.55	88.89	35.83	*Sq* (µm)	0.012	0.011	0.017	0.013	0.003
*Rp* (nm)	39.24	19.61	19.01	25.96	11.51	*Ssk*	−2.492	0.04	1.537	−0.305	2.037
*Rv* (nm)	31.41	26.1	32.69	30.06	3.49	*Sku*	51.17	5.26	18.87	25.10	23.58
*Rc* (nm)	25.08	18.20	16.77	21.64	4.87	*Sv* (µm)	0.291	0.124	0.254	0.223	0.088
*Rq* (nm)	10.87	8.05	7.82	8.91	1.70	*Sz (*µm)	0.603	0.31	0.547	0.487	0.156
*Rsk*	0.344	−0.364	−0.624	−0.21	0.50	-	-	-	-	-	-
*Rku*	6.96	4.3	6.04	5.76	1.35	-	-	-	-	-	-

**Table 2:** Parameters of the shaft material.

ISO	EN	C (%)	Mn (%)	Si (%)	P (%)	S (%)	Mechanical Properties			
							Tensile Strength (MPa)	Yield Strength (MPa)	Elongation (%)	Hardness HB
1.1213	C53	0.50–0.57	0.40–0.70	0.15–0.35	max 0.025	max 0.035	≥630	≥375	≥14	No heat treatment ≤ 241 annealed ≤ 207

**Table 3:** Specifications of the machining parameters for hard turning and ball burnishing.

Hard Turning					Ball Burnishing		
Feed Rate *ft* (mm/rev)	Depth of Cutting *ap* (mm)	Cutting Speed *vc* (m/min)	Code		Feed Rate *fb* (mm/rev)	Burnishing Speed *vb* (m/min)	Code
0.10	0.3	154	HT1		0.06	140	B1
0.18	0.3	154	HT2		0.20	140	B2

**Table 4:** Results of surface machining as profile roughness parameters and hybrid parameters.

2D Roughness Parameters		Machining Sequence					
		HT1	HT2	HT1+B1	HT1+B2	HT2+B1	HT2+B2
Ra (arithmetic mean deviation)	(µm)	0.859	1.457	0.416	0.451	0.716	0.749
Rq (root mean squire deviation)	(µm)	1.094	1.766	0.509	0.569	0.830	0.866
Rsk (skewness)	-	0.672	0.094	0.238	−0.279	−0.119	−0.003
Rku (kurtosis)	-	2.979	2.172	2.759	3.076	2.053	1.932
Rz (maximum height of the profile)	(µm)	5.269	8.412	3.481	3.765	4.609	4.656
Rp (maximum profile peak height)	(µm)	3.193	4.471	2.148	1.768	2.182	2.304
Rv (maximum depth of valleys)	(µm)	2.076	3.940	1.333	1.997	2.426	2.352
Rk (core roughness depth)	(µm)	2.563	4.494	1.693	1.449	2.433	2.827
Rpk (reduced peak height)	(µm)	2.295	1.235	0.617	0.504	0.425	0.523
Rvk (reduced valley depth)	(µm)	0.517	1.980	0.713	0.699	0.975	0.442
Mr1 (upper material ratio)	(%)	12.843	14.812	9.483	10.745	5.114	6.279
Mr2 (lower material ratio)	(%)	91.308	88.323	92.320	84.627	90.165	97.567

**Table 5:** Results of surface machining as the area roughness parameters and the area hybrid parameters.

3D Roughness Parameters		Machining Sequence					
		HT1	HT2	HT1+B1	HT1+B2	HT2+B1	HT2+B2
Sa (arithmetic mean deviation of the area)	(µm)	0.849	1.522	0.416	0.446	0.728	0.755
Sq (root mean square deviation of the area)	(µm)	1.079	1.856	0.513	0.555	0.859	0.867
Ssk (skewness of the area)	-	0.669	0.089	0.592	−0.171	−0.056	0.089
Sku (kurtosis of the area)	-	3.433	2.611	10.386	2.801	2.496	1.923
Sz (maximum height of the area)	(µm)	30.547	14.434	24.153	5.350	12.850	9.931
Sp (maximum peak height of the area)	(µm)	13.084	9.233	18.462	2.556	9.847	2.957
Sv (maximum pit valley height of the area)	(µm)	17.463	5.201	5.691	2.794	3.003	6.974
Sk (core height of the area)	(µm)	2.319	4.653	1.438	1.401	2.698	2.540
Spk (reduced peak height of the area)	(µm)	1.676	1.112	0.480	0.433	0.394	0.492
Svk (reduced valley height of the area)	(µm)	0.620	1.873	0.302	0.549	0.480	0.302
SMr1 (upper material ratio of the area)	(%)	17.882	16.442	8.891	9.291	3.996	10.290
SMr2 (lower material ratio of the area)	(%)	91.908	89.873	93.407	87.612	93.806	97.003
